# Examining the Quality of Life and Discrimination Impact on Parents of Children With Autism Spectrum Disorder in Aseer Region, Saudi Arabia: A WHO-QOL Survey

**DOI:** 10.7759/cureus.53616

**Published:** 2024-02-05

**Authors:** Hayfa A AlHefdhi, Ahmed S AL Zomia, Nawaf M Alshehri, Abdullah A Alaskari, Abdulaziz A Hussain, Lama A Lahiq, Muzun A Asiri, Wahid Al asiri, Abdullah M Alahmari, Hamad M Asiri, Sultan A Alomari

**Affiliations:** 1 Family and Community Medicine, King Khalid University, Abha, SAU; 2 College of Medicine, King Khalid University, Abha, SAU; 3 Medicine and Surgery, King Khalid University, Abha, SAU; 4 Emergency Department, King Fahad General Hospital, Jeddah , SAU

**Keywords:** mental disorders child development, mental disorders, stigma and discrimination, quality of life, saudi arabia, autism

## Abstract

Background

Autism spectrum disorder (ASD) is a neurodevelopmental disease marked by social and repetitive or restricted behaviors, as well as communication difficulty.

Objectives

This survey aimed to assess the quality of life (QoL) of parents with ASDs in the Aseer region of Saudi Arabia using the brief form of the World Health Organization (WHO-QOL) questionnaire. Furthermore, we sought to measure the severity of discrimination experienced by parents of children with ASDs and their impact on QoL.

Methodology

Using a Google form, a cross-sectional study was carried out online between March and April 2023. The patient records from four different regions of Saudi Arabia were used to recruit study participants. The survey was distributed through well-known social media channels (Instagram, Telegram, Facebook).

Results

A total of 99 parents were included in this study. The Southern region accounted for the bulk of participants (81.8%, n=81), nearly three-fourths of the children were boys (70.7%), mothers were more common among respondents (65.7%, n=65) than fathers, 66.7% of respondents reported being married, and 78.8% fall into the middle economic class category. The main source of information among the studied population was the Internet (39.4%, n=39), followed by relatives (23.0%, n=23), physicians (8.1%, n=9), and finally books (4%, n=4). The mean scores for the various domains are as follows: physical (58.48 ± 13.84), psychological (62.04 ± 18.08), social relations (61.20 ± 23.24), environment (24.12 ± 14.62), general QoL (72.93 ± 4.30), and general health (73.94 ± 4.63). Nearly half (46.5%) of parents have encountered stigma or discrimination toward their child or family. Individuals who reported experiencing discrimination exhibited significantly lower mean scores in multiple QoL domains than those who did not report discrimination for physical (54.11, ± 14.36vs, 62.26±12.28, p=0.003), psychological (55.80 ± 20.33 vs 67.45 ± 13.94, p=0.002), and social relations (55.43± 24.17 vs 66.20 ± 21.40, p=0.022). Multivariate analysis revealed that discrimination was the only significant predictor of QoL (p < 0.001).

Conclusions

The QoL of parents having a child with autism is low, moreover, the coincidence of discrimination and stigma significantly lowered QoL.

## Introduction

Autistic spectrum disorder (ASD) is a neurodevelopmental disease marked by social and repetitive or restricted behaviors, as well as communication difficulty [1]. ASD develops in infancy and persists into adolescence and maturity. In most circumstances, during the first five years of life, the criteria are obvious. People with ASD frequently have several co-occurring disorders such as anxiety, depression, attention deficit hyperactivity disorder (ADHD), and epilepsy. The intellectual level of functioning in people with ASDs varies greatly, going from severe to higher levels of disability [2]. One in 700 to 1000 people have ASD and one in 1000 people exhibit the typical signs of autism. Three to four boys are affected by ASD for every girl worldwide [3,4]. It encompasses five clinical subtypes under the term autism spectrum disorder, including Asperger syndrome, autistic disorder, pervasive developmental disorder, Rett syndrome, and childhood disintegrative disorder; it has an impact on social, emotional, and cognitive abilities. It has been suggested that changes in several genes along with environmental circumstances are what causes the autism phenotype to develop; nevertheless, the precise etiologies and neurological basis of autism remain largely unclear [3,5].

Its behavioral symptoms, which can range in severity and affect cognitive function, include early disruption of social connection and communication, restriction, recurrent loss of interest in different activities, and stereotyped behavior patterns [6]. The most typical co-morbidities include aggressiveness, self-injury, adaptive function impairment, and sensory processing problems. Clinically, the fifth edition of the Diagnostic and Statistical Manual of Mental Disorders (DSM-5) is typically used to make a diagnosis of the central primary symptoms [7].

According to the literature, a parent's ability to support their child's general welfare and emotional health may be impacted by their emotional health. Parental stress, for instance, can deplete both parents' and children's coping mechanisms and impair both parties' capacity for problem-solving [8]. Concerns about parental well-being have been highlighted by research on the difficulties associated with raising a kid with ASD. This demographic has been linked to several similar problems, including poor mental and physical health, social isolation, and broken families [9,10]. While earlier research in this area has only emphasized the burdens and demands of caregiving such as stress [11], recent research has focused on parental quality of life (QoL) in an effort to provide a more thorough evaluation of parental adaptation. The concept of QoL is complicated and multifaceted, enabling a thorough assessment of adaptation - both beneficial and detrimental - across numerous functional domains. QoL is defined as "an individual's view of their place in life in relation to their objectives, expectations, standards, and worries, as well as the culture and value systems in which they live" [12]. Studies looking at the QoL of parents of children with ASD have mostly painted a bleak image. A systematic review of the QoL of parents with children with ASD revealed that parents have lower QoL than parents of children who are usually developing [13].

Stigma and discrimination also play a primary obstacle to improving the quality of care and accessibility of mental health services. It was identified as a significant hurdle preventing the integration of individuals living with mental disorders into community activities, healthcare provisions, workplaces, and educational establishments [14]. The negative impact of autism stigma on well-being includes compromised mental and physical health, weakened social connections, and increased efforts to conceal autistic traits. Family members also experience stigma, affecting their well-being. To address this issue, creating inclusive "autism-friendly" environments, promoting positive media portrayals, improving public and professional understanding through enhanced autism education, and supporting neurodiversity are essential steps in reducing societal stigma [15].

There hasn’t been much research done in Saudi Arabia that has focused on the QoL of parents of children with ASDs. In a 2012 study that compared the careers of children with ASDs to those of children with other disabilities, it was discovered that caregivers of families with children with ASDs had the lowest QoL levels [16]. In a 2016 study conducted in Riyadh, researchers compared the QoL of parents of children with and without ASDs and discovered statistically significant disparities between the groups' QoL scores and their relative QoL outcomes [17]. This survey aimed to assess the QoL of parents with ASDs in the Aseer region of Saudi Arabia using the brief form of the WHO-QOL questionnaire [18]. Additionally, we sought to measure the severity of discrimination experienced by parents of children with ASDs and its impact on QoL.

## Materials and methods

Study setting and study design

Using a Google form, a cross-sectional study was carried out online between March and April 2023. Patients' records from four different regions of Saudi Arabia were used to recruit study participants. The survey was distributed through well-known social media channels (Instagram, Telegram, and Facebook).

Study population

Using G 3.1.9, an alpha error of 0.05, power of 95%, and size effect of 0.5, the minimum required sample size was 54. We increased the sample size to 100 to compensate for incomplete data and a non-response rate of 50%. Parents of children who had an ASD diagnosis were included. Parents who were 18 years of age or older and voluntarily volunteered to participate were included in our study. People with communication issues or those without access to a smartphone and the Internet were excluded.

Study questionnaires and data collection

Sociodemographic information was gathered from the participants' responses, including their geographic region (Eastern, Middle, Northern, Southern), relationship to the child (father, mother), marital status (married or single, separated or divorced), education level (primary, secondary, high school, collegiate), economic status (low, medium, high), occupation (business, engineering, housewife, other, teacher, unemployed), and the number of children (four or more children, one child, three children, two children). We used the validated Arabic short version of the World Health Organization Quality of Life (WHOQOL-BREF) instrument to measure QoL in our study [19]. This tool has four areas that cover the physical, psychological, social, and environmental facets of QoL. The 26-item instrument measures QoL across the four previously indicated domains: physical (7 items), psychological (6 items), social connections (3 items), and environmental (3 items). Two items assess general QoL and general health (8 items). To evaluate QoL using the WHOQOL-BREF, participants must respond on a Likert scale with a maximum score of 5. One is used to indicate options like "very poor," "very unsatisfied," "none," or "never," while five is used to represent options like "very good," "very satisfied," "very," or "always." The domain scores were calculated after data collection by adding the individual item scores for each domain. Then, these domain ratings were converted to a positive 0-100 scale, where higher scores indicate a higher QoL [20].

Ethical approval and consent

All participants received thorough information about the study's objectives and purpose, giving them the freedom to choose whether or not to give their informed consent and take part in the study. The research team placed a strong priority on preserving the privacy and anonymity of participant data throughout the whole research procedure. Following the guidelines of the Declaration of Helsinki, ethical approval was acquired by the University of King Khalid Research Ethics (ECM#2023-2125).

Statistical analysis

Statistical analysis was carried out using R software version 4.1.1 (R Core Team (2021). R Foundation for Statistical Computing, Vienna, Austria). Categorical variables were expressed as frequencies and percentages. Numerical variables were presented using mean and SD. We employed the t-test to assess the differences in mean scores for various domains of QoL between two distinct groups: individuals who reported experiencing discrimination and those who did not. We employed an analysis of covariance (ANCOVA) model to examine the relationship between several key independent variables and QoL domains.

Independent Variables

We considered multiple independent variables, including discrimination, relationship to the child, parent gender, child gender, and education level. We used a Type III Sum of Squares analysis to determine the impact of these variables on QoL scores. A p-value below 0.05 was considered statistically significant for this analysis.

## Results

The Southern region accounted for the bulk of participants (81.8%, n=81), with less participation from the Eastern, Middle, and Northern regions. Nearly three-fourths of the children were boys (70.7%, n=70), and 29.3% were girls (n=29). Mothers were more common among respondents (65.7%) than fathers when comparing the gender of the parents (34.3%). Sixty-six point seven percent (66.7%) of people reported being married while 33.3% reported being single. In terms of education, those with a college degree outnumber those without (65.7%, n=65), and the majority of them fall into the middle economic class category (78.8%, n=78). Among various occupations, teachers constitute a significant portion (37.4%, n=37) while the distribution of the number of children indicates diversity, with the highest percentage (45.5%, n=45) having four or more children (Table 1).

**Table 1:** Sociodemographic characteristics of parents of children with autism

Studied variable overall (N=99)		N (%)
Region	Eastern	4 (4.0%)
	Middle	10 (10.1%)
	Northern	4 (4.0%)
	Southern	81 (81.8%)
Child gender	Boy	70 (70.7%)
	Girl	29 (29.3%)
Respondent	Father	34 (34.3%)
	Mother	65 (65.7%)
Marital status	Married	66 (66.7%)
	Single separated or divorced	33 (33.3%)
Education level	Primary	4 (4.0%)
	Secondary	4 (4.0%)
	High school	26 (26.3%)
	Collegiate	65 (65.7%)
Economic status	Low	9 (9.1%)
	Medium	78 (78.8%)
	High	12 (12.1%)
Occupation	Business	5 (5.1%)
	Engineering	4 (4.0%)
	Housewife	13 (13.1%)
	Teacher	37 (37.4%)
	Other	33 (33.3%)
	Unemployed	7 (7.1%)
Number of children	One child	24 (24.2%)

About 53.5% (n=53) of parents have noticed developmental differences in their children compared to their peers while 46.5% (n=46) have not. Approximately 35.4% (n=34) of parents have attended workshops related to neurodevelopmental disorders while 64.6% have not. Almost three-quarters (75.8%, n=75) of parents knew someone who was undergoing treatment for autism spectrum disorder. Sixty-two point six percent (62.6%; n=62) of children were diagnosed with autism spectrum disorder between the ages of one and five years. Various social behavior problems were reported, with social isolation/peer sharing being the most prevalent (35.4%, n=34). Approximately 57.6% (n=57) of parents feel that there was not enough information available to parents on neurodevelopmental disorders. Nearly half (46.5%, n=46) of parents have encountered stigma or discrimination toward their child or family. Sixty-two point six percent (62.6%; n=62) of parents believed that schools and other institutions were not adequately equipped to support children with neurodevelopmental disorders. About 44.4% of parents have sought therapy or other interventions for their child's neurodevelopmental disorder. Nearly two-thirds (60.6%, n=60) of parents have spoken to other parents who have children with neurodevelopmental disorders to share experiences and provide support, and 68.7% (n=68) of parents believed that society, as a whole, is becoming more aware and accepting of neurodevelopmental disorders (Table 2).

**Table 2:** Parental experiences and perceptions of neurodevelopmental disorders in children

Studied variables (N= 99)		N (%)
Child characteristics		
Child gender	Boy	70 (70.7%)
	Girl	29 (29.3%)
‏When was the child diagnosed with autism spectrum disorder?	1 – 5 years	62 (62.6%)
	6 – 10 years	27 (27.3%)
	11 – 15 years	8 (8.1%)
	16 – years	2 (2.0%)
Have you ever noticed any developmental differences in your child compared to their peers?	No	46 (46.5%)
	Yes	53 (53.5%)
Parents' perception and knowledge		
	Do you think that schools and other institutions are adequately equipped to support children with neurodevelopmental disorders?	No	62 (62.6%)
Yes		37 (37.4%)
Do you know anyone who is undergoing treatment for autism spectrum disorder?	No	24 (24.2%)
	Yes	75 (75.8%)
Have you ever sought out therapy or other interventions for your child's neurodevelopmental disorder?	No	55 (55.6%)
	Yes	44 (44.4%)
Have you spoken to parents about neurodevelopmental disorders?	No	39 (39.4%)
	Yes	60 (60.6%)
Social behavior problems	Aim to distinguish between friends and strangers	5 (5.1%)
	Avoiding/ignoring other children	5 (5.1%)
	Difficulty picking up on social cues	15 (15.2%)
	Difficulty understanding feelings	9 (9.1%)
	other	11 (11.1%)
	Problems playing with other children	9 (9.1%)
	Social and personal boundaries	10 (10.1%)
	Social isolation/peer sharing	35 (35.4%)
Do you feel that there is enough information available to parents?	No	57 (57.6%)
	Yes	42 (42.4%)
Attend workshop	No	64 (64.6%)
	Yes	35 (35.4%)
Do you think that society as a whole is becoming more aware and accepting of neurodevelopmental disorders?	No	31 (31.3%)
	Yes	68 (68.7%)
Have you ever spoken to other parents who have children with neurodevelopmental disorders to share experiences and support?	No	39 (39.4%)
	Yes	60 (60.6%)
Discrimination		
Have you ever encountered any stigma or discrimination towards your child or family?	No	53 (53.5%)
	Yes	46 (46.5%)

**Figure 1:**
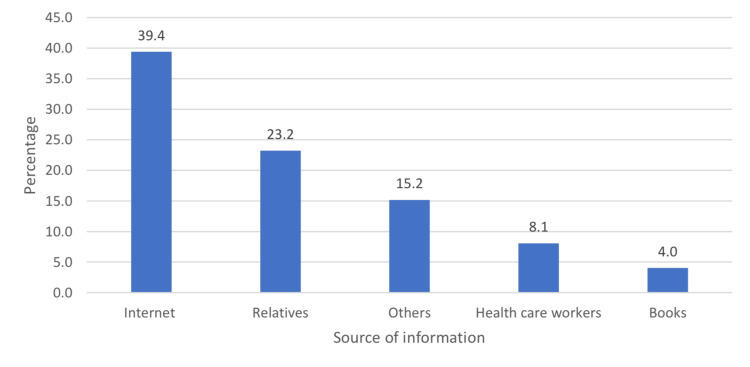
The source of information about autism

Figure 1 shows that the main source of information among the studied population was the Internet (39.4%, n=39), followed by relatives (23.0%, n=23), physicians (8.1%, n=9), and finally books (4%, n=4).

**Figure 2:**
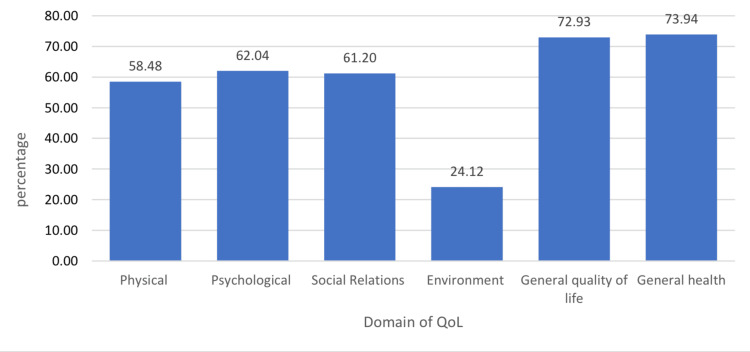
The mean score of the QoL domains QoL: quality of life

The mean scores for the various domains are as follows: Physical (58.48 ± 13.84), Psychological (62.04 ± 18.08), Social Relations (61.20 ± 23.24), Environment (24.12 ± 14.62), General QoL (72.93 ± 4.30), and General Health (73.94 ± 4.63) (Figure 2).

In comparing the two groups, individuals who reported experiencing discrimination exhibited significantly lower mean scores in multiple QoL domains than those who did not report discrimination. Specifically, in the "Had Discrimination" group, mean scores for the Physical (54.11 ± 14.36), Psychological (55.80 ± 20.33), and Social Relations (55.43±24.17) domains were significantly lower compared to the "No Discrimination" group (62.26 ± 12.28, 67.45 ± 13.94; 66.20 ± 21.40, respectively), with p-values of 0.003, 0.002, and 0.022, respectively, indicating statistical significance. However, there were no statistically significant differences between the groups in the Environment, General QoL, and General Health domains, with p-values of 0.122, 0.060, and 0.067, respectively (Table 3).

**Table 3:** Quality of life scores of the parents of children with ASD QoL: quality of life; ASD: autism spectrum disorder

QoL domain	Had discrimination		No discrimination		t	df	p
	mean	sd	mean	sd			
Physical	54.11	14.36	62.26	12.28	-3.043	97	0.003
Psychological	55.80	20.33	67.45	13.94	-3.277	77.993	0.002
Social Relations	55.43	24.17	66.20	21.40	-2.33	90.723	0.022
Environment	21.67	15.75	26.24	13.35	-1.562	97	0.122
General quality of life	3.41	1.33	3.85	0.86	-1.906	75.387	0.060
General health	3.46	1.39	3.91	0.93	-1.859	76.350	0.067

The overall model is statistically significant (F=44.650, p < 0.001), indicating that it explains a significant portion of the variance in the dependent variable, "QoL domains." The model explained a substantial amount of the variance in QoL (R squared = 0.480), suggesting that the factors included in the analysis have a meaningful influence on this dependent variable. The intercept was highly significant (p < 0.001), suggesting that the model's intercept, which represents the baseline value of “QoL," significantly differs from zero. Relationship to the child does not have a significant effect on QoL scores (p=0.191). Both the gender of parents and "Child gender" do not show significant effects on QoL (p=0.415 and p=0.885, respectively). Education level also did not have a significant impact on QoL (p=0.958). Discrimination has a highly significant effect on QoL (p < 0.001), indicating that it plays a crucial role in explaining the variation in the dependent variable. There was a significant difference across the four domains (p <0.001) (Table 4). 

**Table 4:** Multivariate analysis of the factors affecting QoL R2=48 QoL: quality of life

Dependent Variable: QoL domains	Mean Square	F	Sig.	Partial Eta Squared
Corrected model	13441.921	44.650	.0001	.480
Intercept	180697.109	600.223	.0001	.608
Discrimination	6849.364	22.752	.0001	.056
Relationship to the child	516.479	1.716	.191	.004
Parent gender	200.791	.667	.415	.002
Child gender	6.269	.021	.885	.000
Education level	.844	.003	.958	.000
Type	33118.211	110.009	.0001	.460

A post hoc analysis revealed that there were statistically significant differences between the Physical and Environmental domains (p=0.0001) and between the Social and Environmental domains (p=0.0001) (Table 5). 

**Table 5:** The post hoc analysis of the difference between the domains of QoL of parents with autistic children * statistically significant QoL: quality of life

Domains of QoL	Mean Difference	p
Physical vs psychological	3.56	.966
Physical vs social relations	2.72	.999
Physical vs environmental	34.36^*^	.0001
Psychological vs social relations	0.84	.999
Psychological vs environmental	37.92^*^	.0001
Social relations vs environmental	37.08^*^	.0001

## Discussion

In this study, our main objectives were to evaluate the QoL of parents raising children with ASD and to measure the extent of discrimination they face. Additionally, we examined the QoL scores within two distinct groups: those who have experienced discrimination and those who have not. Lastly, we investigated the primary sources of information that parents rely on to acquire knowledge about their children's condition.

Since autism presents so many difficulties for the family, parents of children with autism children experience lower QoL than parents of children with other developmental problems. Parental QoL, which is influenced by numerous parental and child factors, is crucial for children's well-being [13]. Our findings revealed that parents raising children with autism experienced notably lower QoL scores. The environmental domain garnered the lowest score, closely followed by the physical domain, whereas the highest scores were observed in the general health and general QoL domains, with values of 73.94 and 72.93, respectively. A substantial portion of the participants, accounting for 46.5%, reported encountering discrimination and stigma. Interestingly, all QoL domains exhibited lower scores among those who reported experiencing discrimination. Notably, significant differences were observed in the physical, psychological, and social domains, indicating a more pronounced impact of discrimination on these aspects of QoL. Using the ANCOVA model, discrimination was the only significant predictor of lower scores of the QoL of parents.

Many studies conducted worldwide proved lower QoL scores among parents of children with autism [21-24]. Kuhlthau et al. [21] examined the health-related quality of life (HRQoL) of parents of children with ASD using mixed methods. Findings showed slightly worse HRQoL scores, particularly in stress and mental health, and 40% of parents had clinical depression symptoms. Married parents report lower depression symptoms. Alwahabi and her colleagues reported that Saudi moms of disabled children are less satisfied than mothers of typically developing children on the social and economic subscale (QLI Part 1) (p=0.0068) [25]. Raju et al. studied QoL in parents of children with ASD in India and its relationship with socio-demographic factors [20]. They used a questionnaire and WHOQOL-BREF instrument, finding significant differences in QoL between parents of normal children and those who had children with ASD, and reported a positive correlation between socio-demographic variables and QoL. Al-Jabri et al. conducted a cross-sectional study in Jeddah from May to July 2019 to assess the impact of caring for children with ASD on QoL [23]. They found that caregivers with ASDs scored lower in most domains. A study involving 84 parents of children with autism in Arar, Saudi Arabia, found that 63.1% of caregivers had impaired QoL. The main issues were energy/fatigue and emotional limitations. Factors such as female gender, unemployment, and low income were associated with poor QoL. Children with autism of the first birth order and those with a long duration of the disease were more likely to have poor parental QoL. However, gender, income, occupation, and illness duration were not statistically significant [24]. It is worth noting that the coronavirus disease 2019 (COVID-19) negatively affected the QoL of the general population [26,27]. This effect extended to the parents of healthy children and children with disabilities as well as reported in a study conducted in Saudi Arabia recruiting 340 mothers. The QoL was affected in both, however, the delirious effect was observed more among parents of children with disabilities [28].

Moreover, autism may have a more negative impact on parents' QoL than other neurodevelopmental illnesses like attention deficit hyperactivity disorder (ADHD). Compared to families of children with ADHD or unaffected controls, families of children with autism reported more significant negative effects on QoL [22]. A study of 261 Saudi caregivers of Down syndrome children reported higher WHOQOL-BREF domain scores (physical 84 (±15), psychological 88 (±15), social relations 41 (±10), and environmental domains 105 (±24). Educational level and number of children are significantly associated with the Psychological and Physical domains, and the number of children is the only significant variable for social relations [29].

The severity of the core ASD characteristics, the presence of co-morbid conditions, particularly maladaptive behaviors like hyperactivity, oppositional defiant disorder, conduct issues, anxiety, and emotional symptoms, as well as the degree of general developmental delay and impairment in daily living activities, have all been identified as having an impact on parental QoL [30]. We studied a disregarded issue, stigma and discrimination, which had an impact on QoL. Autism is seen as an identity-based minority, and it is generally known that minority groups endure stigma [15]. The unique experience of stigma for people with autism, however, has received relatively little research, with a bias toward family members' experiences rather than those of people with autism themselves [31]. This may explain the lower QoL scores among parents who experience stigmatization.

Strengths and limitations

It's worth noting that this study stands out as one of the few in Saudi Arabia addressing the quality of life among parents of children with autism. However, it comes with certain limitations such as the use of non-random sampling techniques and participant recruitment through social media. Importantly, recent studies indicate widespread use of social media platforms among large segments of the Saudi population, which helps mitigate the risk of selection bias [32].

## Conclusions

In conclusion, our study sheds light on the QoL of parents raising children with ASD and the prevalence of discrimination within this population. Our findings underscore the substantial challenges faced by these parents, as they reported notably lower QoL scores across various domains. The environmental and physical domains emerged as areas of particular concern that warranted targeted support and interventions. Conversely, the relatively higher scores in the general health and general QoL domains indicate potential areas of resilience and well-being among this group. The significant prevalence of discrimination and stigma among the participants is a concerning aspect of our study, affecting nearly half of the respondents. This underscores the need for increased awareness and efforts to combat discrimination against parents of children with ASD. Additionally, our study highlights that discrimination disproportionately impacts the physical, psychological, and social aspects of QoL, underscoring the urgent need for targeted support and advocacy to improve the overall well-being of this population. Overall, our findings provide valuable information on the multifaceted challenges faced by these parents, serving as a foundation for further research and initiatives aimed at improving their QoL and addressing the issue of discrimination.

## References

[1] Lord C, Elsabbagh M, Baird G, Veenstra-Vanderweele J (2018). Autism spectrum disorder. Lancet.

[2] Alnusayri Alnusayri, A.M.F A.M.F (2021). Autism spectrum disorders (ASD) diagnosis and management; current trends and future direction: a review article. Int J Clin Exp Med.

[3] Yoo H (2015). Genetics of autism spectrum disorder: current status and possible clinical applications. Exp Neurobiol.

[4] Loomes R, Hull L, Mandy WP (2017). What is the male-to-female ratio in autism spectrum disorder? A systematic review and meta-analysis. J Am Acad Child Adolesc Psychiatry.

[5] Oviedo N, Manuel-Apolinar L, de la Chesnaye E, Guerra-Araiza C (2015). Genetic and neuroendocrine aspects in autism spectrum disorder [Article in Spanish]. Bol Med Hosp Infant Mex.

[6] Hodges H, Fealko C, Soares N (2020). Autism spectrum disorder: definition, epidemiology, causes, and clinical evaluation. Transl Pediatr.

[7] Fabiano F, Haslam N (2020). Diagnostic inflation in the DSM: a meta-analysis of changes in the stringency of psychiatric diagnosis from DSM-III to DSM-5. Clin Psychol Rev.

[8] Friedman RJ, Chase-Lansdale PL (2002). Chronic adversities. Child and Adolescent Psychiatry.

[9] Giallo R, Wood CE, Jellett R, Porter R (2013). Fatigue, wellbeing and parental self-efficacy in mothers of children with an autism spectrum disorder. Autism.

[10] Drogomyretska K, Fox R, Colbert D (2020). Brief report: stress and perceived social support in parents of children with ASD. J Autism Dev Disord.

[11] Hastings RP, Taunt HM (2002). Positive perceptions in families of children with developmental disabilities. Am J Ment Retard.

[12] Group TW (1998). The World Health Organization quality of life assessment (WHOQOL): development and general psychometric properties. Soc Sci Med.

[13] Vasilopoulou E, Nisbet J (2016). The quality of life of parents of children with autism spectrum disorder: a systematic review. Res Autism Spectr Disord.

[14] Knaak S, Mantler E, Szeto A (2017). Mental illness-related stigma in healthcare: barriers to access and care and evidence-based solutions. Healthc Manage Forum.

[15] Turnock A, Langley K, Jones CR (2022). Understanding stigma in autism: a narrative review and theoretical model. Autism Adulthood.

[16] Haimour AI, Abu-Hawwash RM (2012). Evaluating quality of life of parents having a child with disability. Int Interdiscip J Educ.

[17] Asi KY (2016). Quality of life among parents of children with autism spectrum disorder in Riyadh. International Research in Education.

[18] Group W (1993). Study protocol for the World Health Organization project to develop a quality of life assessment instrument (WHOQOL). Qual Life Res.

[19] Ohaeri JU, Awadalla AW (2009). The reliability and validity of the short version of the WHO quality of life instrument in an Arab general population. Ann Saudi Med.

[20] Group Group, W. W., WHOQOL-BREF WHOQOL-BREF (1996). WHOQOL-BREF: introduction, administration, scoring and generic version of the assessment. https://iris.who.int/bitstream/handle/10665/63529/WHOQOL-BREF.pdf?sequ.

[21] Kuhlthau K, Payakachat N, Delahaye J (2014). Quality of life for parents of children with autism spectrum disorders. Res Autism Spectr Disord.

[22] Raju S, Hepsibah PE, Niharika MK (2023). Quality of life in parents of children with autism spectrum disorder: emphasizing challenges in the Indian context. Int J Dev Disabil.

[23] Al-Jabri BA, Abualhamael RM, Al Hazza MT, Bahabri SA, Alamri YM, Alghamdi BM (2022). Quality of life of caregivers of autistic children in Saudi Arabia: cross-sectional study. Neurosciences (Riyadh).

[24] Alenazi DS, Hammad SM, Mohamed AE (2020). Effect of autism on parental quality of life in Arar city, Saudi Arabia. J Family Community Med.

[25] Alwhaibi RM, Zaidi U, Alzeiby I (2020). Quality of life and socioeconomic status: a comparative study among mothers of children with and without disabilities in Saudi Arabia. Child Care Pract.

[26] Tahoun MM, Ismail HM, Fiidow OA, Ashmawy R, Hammouda EA, Elbarazi I, Ghazy RM (2023). Quality of life among the Arab population two years after COVID-19 pandemic. BMC Public Health.

[27] Ghazy RM, Abubakar Fiidow O, Abdullah FS (2022). Quality of life among health care workers in Arab countries 2 years after COVID-19 pandemic. Front Public Health.

[28] Al Awaji N, Aldhahi M, Akil S, Awad S, Mortada E (2021). Quality of life, needs and fears of mothers of children with disabilities in Saudi Arabia during the COVID-19 lockdown. Int J Environ Res Public Health.

[29] AlAhmari FS, Alageel AF, Aldosari MA, Bagha MY (2022). The quality of life of parents of children with Down syndrome in a tertiary care hospital: a qualitative research study at Saudi Arabia. Ann Med Surg (Lond).

[30] Asahar SF, Malek KA, Isa MR (2021). Quality of life and child’s autism-specific difficulties among Malaysian main caregivers: a cross-sectional study. Int J Environ Res Public Health.

[31] Botha M, Dibb B, Frost DM (2022). "Autism is me": an investigation of how autistic individuals make sense of autism and stigma. Disability & Society.

[32] Alshaikhi OA, Alshaikhi SA, AlZubaidi HA (2023). Social media effect on personal self-esteem among the population in Saudi Arabia. Cureus.

